# [Retracted] Autophagy flux inhibition mediated by celastrol sensitized lung cancer cells to TRAIL-induced apoptosis via regulation of mitochondrial transmembrane potential and reactive oxygen species

**DOI:** 10.3892/mmr.2026.13804

**Published:** 2026-01-19

**Authors:** Uddin Md Nazim, Honghua Yin, Sang-Youel Park

Mol Med Rep 19: 984–993, 2019; DOI: 10.3892/mmr.2018.9757

Following the publication of the above paper, it was drawn to the Editor's attention by a concerned reader that, regarding the cell morphological images shown in Fig. 3A on p. 988, the second panel on the right (showing the Cela −, TRAIL +, CQ + experiment) was strikingly similar in appearance to a data panel that had been included in a paper published by the same research group 3 years earlier in the journal *Oncotarget*, although these data were presented in that article in a different scientific context. Moreover, upon assessing the data in this paper independently in the Editorial Office, there were concerns raised about the possible anomalous appearance of the β-actin blots shown in Fig. 3A, and the Ac-cas3 blot shown in Fig. 3E.

In view of the re-use of the contentious data in the above paper in a different scientific context, and due to the potentially anomalous appearance of some of the western blot data in this paper, the Editor of *Molecular Medicine Reports* has decided that this paper should be retracted from the Journal on account of a lack of confidence in the presented data. The authors were asked for an explanation to account for these concerns, but the Editorial Office did not receive a reply. The Editor apologizes to the readership for any inconvenience caused.

